# CD47 Regulates Collagen I-Induced Cyclooxygenase-2 Expression and Intestinal Epithelial Cell Migration

**DOI:** 10.1371/journal.pone.0006371

**Published:** 2009-07-28

**Authors:** Oliver Jay Broom, Yuan Zhang, Per-Arne Oldenborg, Ramin Massoumi, Anita Sjölander

**Affiliations:** 1 Cell and Experimental Pathology, Department of Laboratory Medicine, Lund University, Clinical Research Centre, Malmö University Hospital (UMAS), Malmö, Sweden; 2 Department of Integrative Medical Biology, Umeå University, Umeå, Sweden; Northwestern University, United States of America

## Abstract

Increased epithelial cell expression of the cyclooxgenase-2 (COX-2) enzyme is a characteristic event of both inflammatory bowel disease and colon cancer. We here report the novel findings that collagen I-induced de novo synthesis of COX-2 in intestinal epithelial cells is inhibited by pertussis toxin (PTX) and by an inhibitory peptide selective for the heterotrimeric Gα_i3_-protein. These findings could be explained by a regulatory involvement of the G-protein-dependent integrin-associated protein CD47. In support of this notion, we observed a collagen I-induced association between CD47 and α2 integrins. This association was reduced by a blocking anti-CD47 antibody but not by PTX or a control anti-β2 antibody. Furthermore, a blocking antibody against CD47, dominant negative CD47 or specific siRNA knock down of CD47, significantly reduced collagen I-induced COX-2 expression. COX-2 has previously been shown to regulate intestinal epithelial cell adhesion and migration. Morphological analysis of intestinal cells adhering to collagen I revealed a co-localisation of CD47 and α2 integrins to non-apoptotic membrane blebs enriched in Rho A and F-actin. The blocking CD47 antibody, PTX and a selective COX-2 inhibitor, dramatically inhibited the formation of these blebs. In accordance, migration of these cells on a collagen I-coated surface or through a collagen I gel were significantly reduced by the CD47 blocking antibody, siRNA knock down of CD47 and the COX-2 inhibitor NS-398. In conclusion, we present novel data that identifies the G-protein-dependent CD47 protein as a key regulator of collagen I-induced COX-2 expression and a promoter of intestinal epithelial cell migration.

## Introduction

The induction of the cyclooxygenase-2 (COX-2) enzyme and the formation of its metabolites, the prostaglandins, are significant and characteristic features of inflammatory responses in general [1], [2]. These include inflammatory intestinal conditions where COX-2 induced formation of prostaglandin E_2_ has been shown to be a regulator not only of inflammatory cells but also of intestinal epithelial cells [2]. COX-2 induction has also been intimately linked to the development and progression of colon cancer and has been shown to be over expressed in colon tumour samples [3]. It has also been suggested that COX-2 plays a crucial role in the well-documented progression from chronic inflammatory bowel disease to colon cancer [4]–[6]. In support of this, selective inhibitors of COX-2 have been shown to significantly reduce the development and progression of colon cancer by reducing tumour cell proliferation and migration [7], [8].

Integrins have been extensively studied because of their essential role in cell attachment to the extracellular matrix, and also for their signalling role in numerous cellular processes, for example cell survival, proliferation, and migration [9]. Integrins have also been implicated in many pathological settings, for example in cancer development [10]–[13]. The signalling capability of integrins can directly or indirectly be regulated by different cellular proteins, such as receptor tyrosine kinases [14] and G-protein coupled receptors [15] including the Integrin Associated Protein also known as CD47 [16]. This protein exhibits varying expression levels in different tissues and has been shown to be up-regulated in ovarian carcinoma cells [17]. The CD47 protein is composed of an extracellular IgV-like domain, five transmembrane spanning domains and a differentially spliced cytoplasmic domain [18], [19]. The signals generated by this protein are known to be coupled to various intracellular signalling pathways through the activation of a *Bordetella pertussis* toxin (PTX) sensitive G-protein [20]. CD47-induced activation of a PTX sensitive G-protein can occur through a *cis* interaction that involves CD47 binding to an integrin. So far the α2β1, α4β1, αvβ3 and αIIbβ3 integrins have been shown to associate with CD47 [21], [22]. Alternatively, CD47-induced activation of a PTX sensitive G-protein can arise through a *trans* interaction i.e. by binding of a specific ligand, for example thrombospondin, directly to CD47 [23].

We have previously shown that collagen engagement of the α2β1integrin on intestinal epithelial cells resulted in increased COX-2 promoter activity and expression of COX-2 [10]. These effects were mediated via α2β1integrin downstream signalling and involved protein kinase Cα, Ras and the transcription factor NFκ B [10]. Increased activity of COX-2 leads to elevated formation of prostaglandins and, in intestinal cells, primarily to an increased generation of prostaglandin E_2_, a lipid mediator that has previously been shown to promote intestinal epithelial cell migration [24], [25]. However, to date, no role has been described for a direct or indirect regulator in the α2β1integrin-induced expression of COX-2 and intestinal epithelial cell migration.

Here we have specifically investigated whether the collagen-induced integrin signal leading to COX-2 expression and intestinal epithelial cell migration requires the transactivation of additional membrane proteins. We present novel data identifying the transmembrane protein CD47 as a key regulator of collagen-induced COX-2 expression and downstream promoter of intestinal epithelial cell migration.

## Methods

### Reagents

The anti-human COX-2 Ab was purchased from AbCam (Cambridge, UK), and the anti-α2 integrin Ab was from Chemicon International (Temecula, CA, USA). The anti-GAPDH and anti-Rho A Abs were from Santa Cruz (Santa Cruz, CA, USA), whereas the anti-actin Ab was obtained from ICN Biomedicals (Temecula, CA, USA). The mouse and rabbit IgG and the anti-β2 integrin Abs as well as the HRP conjugated goat anti-rabbit, and the goat anti-mouse secondary Abs were obtained from Dako (Copenhagen, Denmark). The inhibitory anti-CD47 Ab (B6H12) was purified from hybridoma supernatents by ammonium sulphate precipitation and affinity chromatography, using protein G High Trap columns (Amersham Bioscience, Piscatway, NJ, USA) whereas the anti-CD47 Ab (MEM122) was from EXBIO (Prague, Czech Republic). The dominant negative construct of CD47 [26] was a kind gift from Professor T. Matozaki (Gunma University, Gunma, Japan). The *bordetella pertussis* toxin (PTX) was purchased from Speywood Pharma, Ltd. (Maidenhead, UK). The enhanced chemiluminescence (ECL) reagents and the hyperfilm were from Amersham International (Buckinghamshire, UK). The collagen I and the anti-F-actin Ab for immunostaining were purchased from BD Biosciences (Erembodegem, Belgium) and the thrombospondin-1 (TSP-1) was from Calbiochem (San Diego, CA, USA). The COX-2 specific inhibitor N-(2-cyclohexyl-4-nitrophenyl)methane sulphonamide (NS-398) was purchased from Biomol (Plymouth Meeting, PA, USA).The small inhibitory G-protein peptide minivectors were purchased from Cue Biotech Inc. (Chicago, IL, USA), whilst the lipofectamine 2000, fibronectin and fluorescent secondary Abs Alexa 488, Alexa 568 were from Invitrogen Corp. (Carlsbad, CA, USA). The CD47 siRNAs (ID numbers: 2811, 145978 and 145979) were purchased from Ambion, (Cambridgeshire, UK). All other chemicals were of analytical grade and purchased from Sigma Chemical Co (St Louis, MO, USA).

### Cell culture

Human embryonic intestinal epithelial cells, Int 407 cells [27], exhibiting typical epithelial growth and morphology were cultured as a monolayer to 75–80% confluence for 5 days in Eagle's Basal medium, supplemented with 10% newborn calf serum, 55 µg/ml streptomycin and 55 U/ml penicillin. Cell cultures were regularly tested to ensure the absence of mycoplasma contamination.

### Coating of plates

Dishes (60 mm) were coated with 10 µg/ml collagen I for 1 hour at 37°C, before being washed with PBS, blocked for 30 minutes with 1% BSA at 37°C and then finally washed again with PBS. Control dishes were pre-treated with (3-aminopropyl) triethoxysilane, washed and coated with 6% BSA for 30 minutes at 37°C and washed with PBS.

### Incubation of cells

Serum starved cells (2 hours) were detached and re-suspended in serum free medium. In the indicated experiments these cells were then pre-treated with or without PTX (500 ng/ml) for 2 hours at 37°C. Alternatively, these cells were pre-treated or not with 20 µg/ml of the mouse anti-CD47 functional blocking antibody B6H12, or mouse IgG control or an anti-β2 integrin antibody for 20 minutes at 4°C. These cells were then allowed to adhere to BSA (control) or collagen I coated dishes or slides for 1 hour at 37°C. In some experiments (indicated in figure legend 3) 10 µg/ml of thrombospondin-1 (TSP-1) was added during the 1 hour period of cell adherence to BSA or collagen I coated surfaces.

### Transient transfections

Cells were cultured for 3 days to 50–60% confluence. The cells were then transfected using lipofectamine 2000 with the different Gα-subunit cDNA minigene vectors (coding for specific blocking peptides (cue BIOtech) against the Gα_i1-2_ -, Gα_i3_ –proteins) or with an empty vector. Alternatively, the IgV like domain of CD47 coupled to a GPI anchor constituting a dominant negative CD47 was transfected into the cells using lipofectamine 2000. After 4 hours, the transfection medium was replaced with normal growth medium and the cells were allowed to grow for a further 48 hours before being plated onto collagen I dishes and analysed for COX-2 and CD47 expression as described previously.

### Transfection with CD47 siRNA oligomers

Cells were cultured for 3 days to 50–60% confluence. The cell media was aspirated and the cells detached and scraped into 3 ml of serum and antibiotic free medium containing 50 nM siRNA against CD47 or a scrambled control siRNA with lipofectamine 2000. After 4 hours, the transfection medium was diluted with normal growth medium without antibiotics and the cells were allowed to grow for an additional 48 hours period.

### Immunoprecipitation

The cells were pre-treated as previously described and were allowed to adhere onto collagen I or BSA (control) coated dishes for 1 hour at 37°C. After this period of incubation non-adherent cells were gently washed off with PBS and the remaining cells were lysed (in the lysis buffer described above but with Triton X-100 replaced with 1% octyl-β-D-1-thioglucopyranoside). The lysates were then incubated for 20 minutes on a rotator at 4°C, after which cell debris was removed by centrifugation at 9,000 x g for 10 minutes. Protein G agarose was used to pre-clear the lysates after which the different cell lysates were adjusted to the same protein content. These lysates were then incubated with 20 µg/ml of the anti-CD47 (MEM122) or control IgG antibodies overnight on a rotator at 4°C. Protein G was then added and the lysates incubated at 4°C for 1 hour. After three washes with lysis buffer the final pellets were re-suspended in sample buffer, boiled and analysed by Western blotting as described previously.

### Western blotting

The medium was aspirated and non-adhered cells were removed by washing with ice cold PBS. The cells were then lysed with and scraped loose into an ice-cold lysis buffer (50 mM Tris, pH 7.5, 1 mM EDTA, 1 mM EGTA, 1 mM Na_3_VO_4_, 1% Triton X-100, 50 mM NaF, 5 mM sodium pyrophosphate, 10 mM sodium glycerophosphate, 4 µg/ml leupeptin, and 30 µg/ml phenylmethanesulfonyl fluoride). The resultant lysate was boiled with sample buffer (62 mM Tris pH 6.8, 1.0% SDS, 10% glycerol, 15 mg/ml dithiothreitol, and 0.05% bromphenol blue). Equal amounts of lysed and boiled protein (30–50 µg protein/well) were loaded and subjected to electrophoresis on 8% homogeneous polyacrylamide gels. The separated proteins were electrophoretically transferred to PVDF membranes, which were then blocked for 1 hour at room temperature with either 3% BSA/PBS or 5% non-fat dried milk for Western blotting with the anti-α2 integrin antibody. The membranes were then incubated overnight at 4°C with the primary antibody: anti-COX-2 (1∶500); anti-CD47 (MEM122, 1∶1,000); GAPDH (1∶2,000); α2 integrin (1∶500) or anti-actin (1∶2,000). The membranes were then thoroughly washed and incubated for 1 hour at room temperature with HRP-conjugated secondary antibodies, diluted 1∶3,000 for COX-2, actin and GAPDH; 1∶2,000 for CD47 in 3% BSA/PBS/0.1% Tween-20 and 1∶20,000 for α2 integrin in 5% non-fat dried milk/PBS/0.1% Tween-20. The membranes were then incubated with ECL Western blot detection reagents, and exposed to Hyperfilm-ECL to visualise immunoreactive proteins. Densitometric analysis was performed using a Bio-Rad GS-800 calibrated densitometer, where the value obtained from the control BSA was set as 100.

### Immunofluorescent staining

Cover slips were acid treated with 20% HCl for 1 hour at 60°C, washed with distilled water, treated with 1 M NaOH for 10 minutes, washed and silanised with (3-aminopropyl) triethoxysilane. After extensive washing, 10 µg/ml of collagen I or fibronectin was added (unless otherwise stated) and the cover slips incubated for 1 hour at 37°C. Serum staved cells pre-incubated as described above were plated out on to the coated cover slips for 1 hour. After being washed twice with ice cold PBS, cover slips were fixed for 15 minutes on ice with either 4% ice-cold paraformaldehyde in PBS or with 10% trichloroacetic acid for 15 minutes. The trichloroacetic acid fixed cells were then further fixed for 10 minutes at room temperature in a fixation buffer (137 mM NaCl, 5 mM KCl, 1.1 mM NaH_2_PO_4_, 0.4 mM KH_2_PO_4_, 2 mM MgCl_2_, 2 mM K-EGTA, 5 mM PIPES, pH 6.8, and 5.5 mM Glucose) with 0.5% glutaraldehyde. All cells were then permeabilised with 0.5% Triton X-100 for 5 minutes. The cover slips were then blocked with 3% BSA/PBS solution for 30 min. Thereafter, the cells were incubated at room temperature with primary antibodies against α2 integrin (1∶500), CD47 (1∶300, B6H12), Rho A (1∶100) or F-actin (1∶300) in a 3% BSA/PBS solution for 1 hour. Cells were then washed five times in PBS and incubated with conjugated secondary antibodies (IgG Alexa 488 or IgG Alexa 568) in a 3% BSA/PBS solution for 1 hour. Following five washes with PBS, the cover slips were mounted on glass slides with a fluorescence-mounting medium (Dacon). The mounted slides were examined using a Bio-Rad Radiance 2000 confocal laser scanning system with a Nikon microscope (TE300) or a deconvolution system with a Nikon microscope (TE300).

### Adhesion assay

Samples of 250,000 cells were pre-incubated as previously described and then allowed to adhere onto collagen I coated dishes for 1 hour at 37°C. Following this incubation non-adherent cells were gently washed off with PBS. The remaining cells were then incubated in a physiologically balanced calcium medium containing (4.7 mM KCl, 136 mM NaCl, 1.2 mM MgSO_4_, 1.1 mM EDTA, 1.2 mM KH_2_PO_4_, 5.5 mM glucose, 5 mM NaHCO_3_, 20 mM HEPES; pH 7.4) supplemented with 0.2% nitroblue tetrazolium (NBT) for 2 hour at 37°C after which the cells were placed on ice. Thereafter the NBT solution was aspirated and the cells were fixed in ice cold 70% ethanol for 5 minutes. The cells were then detached and centrifuged at 2,000 xg for 5 minutes. The ethanol containing supernatant was aspirated and 1 ml of N-N-dimethylforamide was added. The suspended pellet was heated for 1 hour at 56°C, then an additional 1 ml of N-N-dimethylforamide was added and left overnight again at 56°C. The solution was once more centrifuged at 2,000 xg for 5 minutes and the optical density of the supernatant was measured at 544 nm.

### Wound healing assay

The cells were pre-incubated as previously described with anti-CD47 and as controls with either an anti-IgG or an anti-β2 integrin antibody for 20 minutes. Where indicated the cells were also pre-incubated with 100 µM of the COX-2 specific inhibitor, NS-398 for 30 minutes on a rotator at 4°C in serum free medium. Cells were then plated onto collagen I coated dishes for 2 hours, thereafter, a sterile pipette tip was used to make a scratch in the cell monolayer and non-adherent cells were removed by gentle washing. The cells were allowed to migrate for 18 hours in serum free medium at 37°C. Measurements of the widths of the wounds were taken at time 0 and 18 hours using the Image J software.

### Cell invasion assay- 3D cell migration

Suspended cells were pre-incubated as described for the wound healing assay or transfected with siRNA oligomers as previously described. Cells (250,000) were added on top of a collagen I containing (3 mg/ml) gel placed in the upper well of a Boyden chamber. The lower well contained serum free medium and was separated from the upper well by a polycarbonate PVPF membrane with 8.0 µm diameter pores. After 18 hours of incubation at 37°C, the cells that were attached to the upper side of the membrane or present in the collagen I gel were removed with a cotton swab, and the remaining cells were fixed with 4% paraformaldehyde, for 15 minutes. The cells in the membrane were subsequently stained with a 1% crystal violet/10% methanol solution at room temperature for 15 minutes. The membranes were washed in PBS after which the remaining dye was solubilised using a 10% SDS solution and the absorbance was measured at 590 nm.

## Results

### Collagen I induced COX-2 expression is regulated by the Gα_i3_ protein

**Figure 1:**
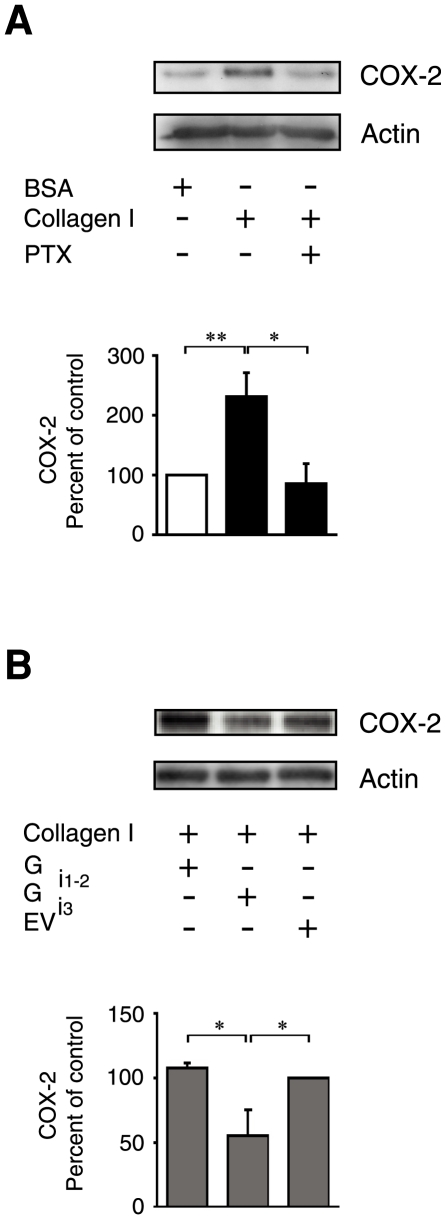
Collagen I induces COX-2 expression through a PTX dependent G-protein. (A) Int 407 cells were incubated with or without 500 ng/ml PTX for 2 hours, after which they were plated out onto 10 µg/ml collagen I or 6% BSA (control) coated dishes for 1 hour. Adherent cells were then lysed and analysed for COX-2 expression by Western blotting as previously described. (B) Cells were transiently transfected with empty vector or vectors expressing small inhibitor peptides against either Gα_i1-2_ or Gα_i3_ before plated onto 10 µg/ml collagen I coated dishes for 1 hour. Adherent cells were the lysed and analysed for COX-2 expression by Western blotting. All membranes were re-probed for actin to ensure equal loading. The accumulated data of the densitometric analyses are given as percent of control and represent means ± SE of four separate experiments. The statistical analyses were performed with unpaired Students t-test; *P<0.05, **P<0.01 relative to the control and compared to collagen I treatment.

In order to address the question of whether the α2β1 integrin-induced COX-2 expression previously reported [10], is also dependent on the transactivation of an additional cell surface G-protein coupled receptor, we first pre-incubated intestinal epithelial cells with PTX. PTX was used as an initial tool to discriminate between integrin and G-protein coupled receptor signalling. The intestinal epithelial cells (Int 407), were allowed to adhere onto a collagen I coated surface for 1 hour, a time period previously shown to result in maximum adhesion [10]. The adhesion to a collagen I coated surface induced a large and statistically significant increase in COX-2 expression, as compared to plating the cells onto a BSA coated surface (Fig. 1A). Interestingly, this collagen I-induced COX-2 expression was abolished by PTX pre-treatment of the cells (Fig. 1A). To further substantiate this finding and in order to identify the specific PTX sensitive G-protein involved, we transfected the cells with plasmids coding for different small peptides that specifically inhibit various Gα_i_ subunits [28]. For the interpretation of the data it is important to note that a transfection efficiency rate of approximately 60% was observed. We observed a 50% reduction in collagen I-induced COX-2 expression in cells transfected with an inhibitory peptide against the Gα_i3_ protein in comparison to cells transfected with an inhibitory peptide against the Gα_i1-2_ protein or empty vector transfected cells (Fig. 1B). Our data thus indicate the presence of a co-regulatory molecule interacting with the Gα_i3_ protein in α2β1 integrin-induced COX-2 expression in these cells.

### The G-protein coupled receptor, CD47, associates with α2 integrins in collagen I adhering intestinal epithelial cells

**Figure 2:**
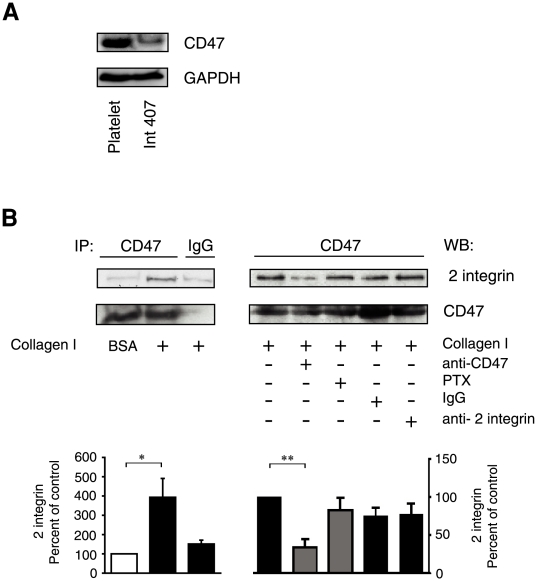
Expression and association of CD47 with α2 integrin upon collagen I stimulation. (A) Whole cell lysates from the intestinal epithelial cell line, Int 407 and platelets, were analysed by Western blotting for their expression of CD47, and re-probed for GAPDH to ensure equal loading. (B) Int 407 cells were pre-incubation with either 500 ng/ml PTX for 2 hours or with either anti-CD47 (B6H12), anti-β2 integrin control antibody or pre-immune control IgG for 20 minutes before being plated onto 10 µg/ml collagen I or 6% BSA coated dishes. The indicated immunoprecipitations, with either an anti-CD47 antibody or control IgG, were performed as outlined in the Methods. Samples were analysed by Western blotting for the presence of CD47 and the α2 integrin. The accumulated data of the densitometric analyses are given as percent of control and represent means ± SE of three separate experiments. The statistical analyses were performed with unpaired Students t-test; *P<0.05, **P<0.01 relative to the control.

Many proteins are known to modulate integrin signalling and function, however only a few have been documented to do this through a PTX sensitive G-protein, one of which is CD47 [16], [23]. We therefore decided to investigate whether CD47 could be responsible for the observed Gα_i3_ protein regulation of collagen I-induced COX-2 expression. Using platelet lysate as a positive control we first confirmed the expression of CD47 in the intestinal epithelial cell line, Int 407 by Western blot (Fig. 2A). Subsequently we detected a significant increase (4-fold) in the amount of α2 integrin recovered in an anti-CD47 immunoprecipitate from cells plated on a collagen I coated surface relative to the immunoprecipitate from cells plated onto the control BSA coated surface (Fig. 2B). An association between CD47 and α2β1 integrins can be taken as an indication that CD47 participates in the regulation of α2β1 integrin signalling and function, as previously demonstrated in vascular smooth muscle cells [21]. Control IgG immunoprecipitates of lysates from cells plated onto collagen I contained a small amount of non-specifically co-immunoprecipitated α2 integrins (Fig. 2B). To further confirm the occurrence of this α2 integrin/CD47 protein-protein interaction we pre-incubated the cells with a functional blocking antibody against CD47 [21], prior to their adherence to collagen I. As expected, the functional inhibition of CD47 resulted in a reduced amount of α2 integrins co-immunoprecipitating with CD47 (Fig. 2B). The observation that pre-incubation with PTX did not affect the collagen I-induced association between CD47 and α2 integrins (Fig. 2B), suggests that the protein-protein interaction occurs prior to CD47-induced G-protein activation. Finally, to confirm the specificity of the anti-CD47 functional blocking antibody we preformed control experiments in which the cells were pre-incubated with either a control IgG or an anti-β2 integrin antibody (a suitable control since endogenous β2 integrin expression is absent in epithelial cells) before plating onto collagen I. The results revealed no effect on the amount of α2 integrins recovered in the anti-CD47 immunoprecipitate (Fig. 2B). We conclude therefore that cell adhesion onto collagen I induces a significant increase in the association between CD47 and α2 integrins that is independent of the activation of a PTX sensitive G-protein.

### CD47 participates in the collagen I-induced expression of COX-2

**Figure 3:**
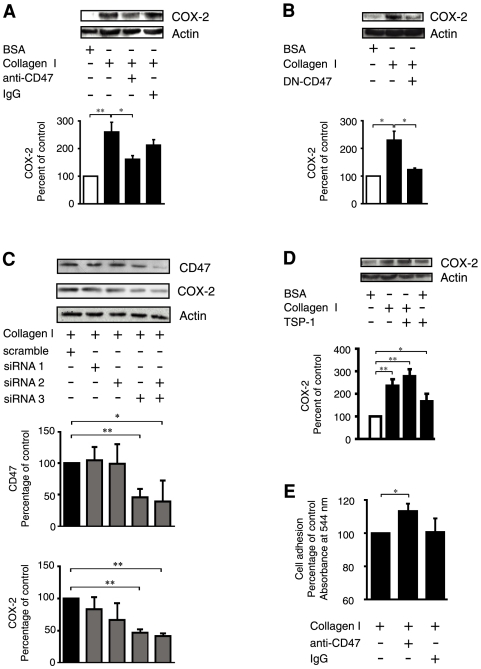
Collagen I mediated COX-2 expression and cell adhesion requires CD47. (A) Int 407 cells were pre-treated with 20 µg/ml of the anti-CD47 functional blocking antibody (B6H12) or control IgG for 20 minutes at 4°C. Whereupon they were plated onto 10 µg/ml collagen I or 6% BSA coated dishes for 1 hour. Adherent cells were lysed and analysed for COX-2 expression by Western blotting as previously described. All membranes were re-probed for actin to ensure equal loading. (B) Cells were transfected with a dominant negative form of CD47 and plated onto 10 µg/ml collagen I or 6% BSA coated dishes for 1 hour. Adherent cells were the lysed and analysed for COX-2 expression by Western blotting. All membranes were re-probed for actin to ensure equal loading. (C) Int 407 cells were transfected with 50 nM siRNA against CD47 or with scrambled control siRNA, for 48 hours. Thereafter cells were plated onto 10 µg/ml collagen I coated dishes for 1 hour. Adherent cells were then lysed and analysed for CD47 and COX-2 expression by Western blotting as previously described. All membranes were re-probed for actin to ensure equal loading. (D) Cells were plated onto 10 µg/ml collagen I, or 6% BSA coated dishes with or without 10 µg/ml TSP-1 for 1 hour. All cells were lysed and the COX-2 expression was analysed by Western blotting. All membranes were re-probed for actin to ensure equal loading. (E) Cell adhesion assay; Int 407 cells were pre-treated with 20 µg/ml of the CD47 functional blocking antibody (B6H12) or control IgG for 20 minutes. Whereupon 250,000 cells were plated onto 10 µg/ml collagen I coated dishes for 1 hour. Adherent cells were measured by their conversion of nitroblue tetrazolium to the insoluble formazan product and the absorbance was measured at 544 nm. The accumulated data are given as percent of control and represent means ± SE of at least five separate experiments. The statistical analyses were performed with unpaired Students t-test; *P<0.05, **P<0.01 relative to the control.

To investigate whether an increased association between α2β1 integrins and CD47 is required for collagen I-induced COX-2 expression, we first pre-incubated the intestinal cells with the functional blocking antibody against CD47 that inhibits this protein-protein interaction (Fig. 2) prior to plating them onto a collagen I coated surface. The blocking antibody significantly reduced collagen I-induced COX-2 expression whereas a control IgG antibody failed to do so (Fig. 3A). Supporting this observation, transfection with a vector containing the extracellular IgV domain of CD47 linked to a GPI membrane anchor and therefore acting as a dominant negative form of CD47 (DN-CD47), resulted in significant inhibition of collagen I-induced COX-2 expression (Fig. 3B). Further to this we also used siRNA, to knock down expression of CD47. One of three specific oligomers (denoted as 1, 2 or 3) or a scrambled control oligomer was transfected into the cells prior to plating them onto a collagen I coated surface. Transfection with the scrambled oligomer, siRNA 1 or siRNA 2, was unable to significantly effect the endogenous level of CD47 (Fig. 3C). However using either siRNA 3 alone or a combination of siRNA oligomers 2 and 3 effectively reduced CD47 expression by approximately 50% (Fig. 3C). Reciprocally, collagen I-induced COX-2 expression was reduced by approximately 60% in cells transfected with siRNA 3 or a combination of siRNAs 2 and 3 (Fig. 3C). As a control experiment we wanted to discern whether CD47 activation alone is capable of inducing COX-2 expression, since this would indirectly support the notion that collagen I-induced expression of COX-2 requires the activation of CD47. For this reason intestinal cells were stimulated with 10 µg/ml thrombospondin-1 (TSP-1), a known activator of CD47 [29]. We found that in cells adhering to a BSA coated surface, TSP-1 stimulation resulted in a statistically significant induction of COX-2 expression (Fig. 3D). On the other hand, cells plated onto collagen I and simultaneously stimulated with TSP-1 only exhibited a slight but statistically non-significant increase in COX-2 expression in comparison with un-stimulated cells plated onto a collagen I coated surface (Fig. 3D). Previously, CD47 and COX-2 signalling have been shown to affect cellular adhesion to extracellular matrix [20], [25]. In order to ensure that our findings obtained with the anti-CD47 inhibitory antibody were not due simply to decreased cell adhesion onto the collagen I coated surface, we tested whether this antibody influenced the adhesion of intestinal epithelial cells. Cells pre-incubated with the anti-CD47 antibody did not have reduced adhesion to collagen I, on the contrary, a significant increase in adhesion was observed. In contrast, pre-incubation with a control IgG antibody did not affect cell adhesion as compared to untreated cells (Fig. 3E). Thus, the effect of the anti-CD47 antibody on collagen I-induced COX-2 expression cannot be explained by decreased adhesion. Instead, the ability of the anti-CD47 antibody to increase adhesion to collagen I suggests that CD47 could be involved in α2β1 integrin mediated intestinal cell migration.

### Localisation of α2 integrin and CD47 to membrane blebs in cells adhering to a collagen I coated surface

**Figure 4:**
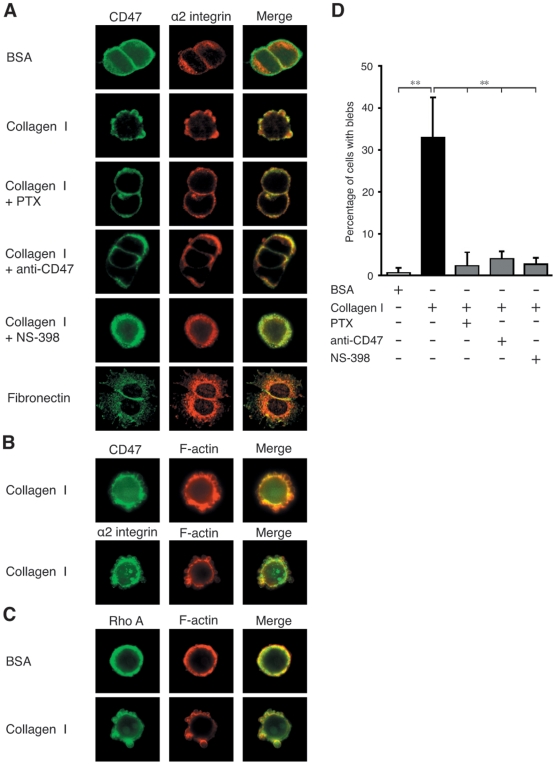
ECM dependent morphological differences modulated through CD47 signalling. (A) Fluorescent microscope images were taken of cells plated onto cover slips coated either with 6% BSA, 10 µg/ml collagen I or fibronectin for 2 hours. Where indicated cells were pre-treated with 500 ng/ml PTX for 2 hours, 20 µg/ml of the CD47 functional blocking antibody for 20 minutes or 100 µM of the COX-2 specific inhibitor NS-398 for 30 minutes. Cells were fixed, permeabilised and stained with primary antibodies against either CD47 or α2 integrin using either Alexa-488 or -546 conjugated secondary antibodies. (B) as in A but stained with primary antibodies against CD47, α2 integrin and F-actin. (C) as in A but stained with primary antibodies against Rho A and F-actin. Shown are representative pictures of three separate experiments taken at 60× magnification. (D) A graphical representation of the number of cells with membrane blebs from 100 cell samples. The statistical analyses were performed with unpaired Students t-test; **P<0.01 relative to the control.

Due to the intriguing finding that CD47 inhibition can increase cellular adhesion to collagen I, a morphological analysis of the cells was performed. We noted that cells, when plated onto glass slides coated with collagen I, exhibited distinct membrane blebs. Furthermore, immunofluoresence analysis revealed co-localisation of CD47 and α2 integrins in these blebs (Fig. 4A). This morphology was distinct from the continuous rounded morphology of the cells plated onto BSA that had a homogenous dispersion of CD47 and α2 integrin around their membranes (Fig. 4A). Pre-incubation with PTX, the functional blocking anti-CD47 antibody or the COX-2 specific inhibitor NS-398, before the cells were plated onto collagen I, produced a similar rounded cell morphology and a homogenously dispersed CD47/α2 integrin staining pattern (Fig. 4A). As a control, we also investigated the adhesion of the cells onto a plates coated with fibronectin, which activates α5β1 and αvβ3 integrins [30]. In stark contrast to the membrane blebbing that characterised adhesion to collagen I, the cells plated onto fibronectin, displayed a well spread morphology with CD47 mainly localised to the areas of cell-cell contacts (Fig. 4A). Immunofluoresence staining analysis of cells plated onto collagen I for CD47, α2 integrins and F-actin showed F-actin co-localisation with CD47 and α2 integrins in these membrane blebs (Fig. 4B). The accumulation of F-actin in the membrane blebs strongly suggests that they are non-apoptotic membrane blebs. Such membrane structures have been previously shown to contain the small GTPase Rho A and are functionally linked to cell migration [31]. In accordance, we also show Rho A localisation in these membrane blebs (Fig. 4C). Quantification of the membrane blebs (Fig. 4D) present in cells confirmed the dependence of these structures on collagen I induced, CD47 mediated COX-2 expression. These experiments convincingly demonstrate that cell adhesion to collagen I induces the formation of non-apoptotic membrane blebs enriched in CD47, α2 integrins, F-actin and Rho A. The formation of these blebs, which are dependent on CD47 mediated collagen induced COX-2 activity, suggested to us the possibility of a functional role for CD47 in intestinal epithelial cell migration.

### CD47 participates in the regulation of collagen I-induced intestinal cell migration

**Figure 5:**
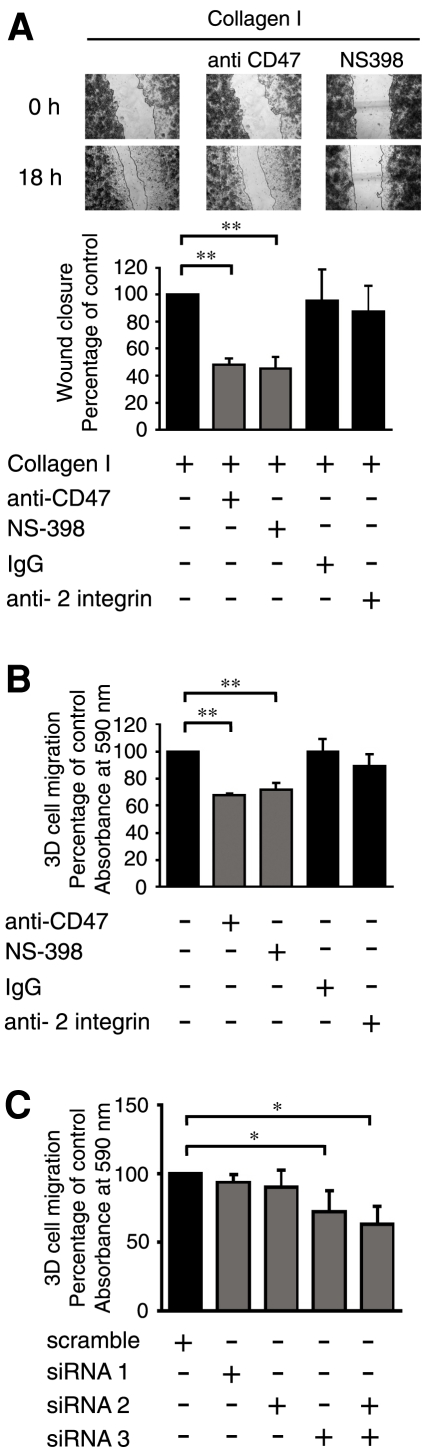
Collagen I dependent 2D and 3D cell migration is regulated by CD47. (A) Wound healing assay; Int cells were pre-incubated or not with 20 µg/ml of the CD47 functional blocking antibody B6H12 for 2 hours, IgG or β2 integrin antibody for 20 minutes, or 100 µM of the COX-2 specific inhibitor NS-398 for 30 minutes, thereafter cells were plated onto 10 µg/ml collagen I coated dishes for 2 hours, after which a wound was made in the monolayer, and the cells were allowed to migrate for 18 hours. Pictures of the wound were taken after 0 and 18 hours. The wound closure was measured and is presented as the percentage of wound closure as compared to time zero. (B, C) 3D cell migration assay; (B) Cells were pre-incubated or not with 20 µg/ml of the CD47 functional blocking antibody B6H12 for 2 hours, IgG or the β2 integrin antibody for 20 minutes, or 100 µM of the COX-2 specific inhibitor NS-398 for 30 minutes after which 250,000 cells from each group were allowed to migrate through a 3 mg/ml collagen I gel and across an 8.0 µm micropore membrane for 18 hours. (C) Int 407 cells were transfected with 50 nM siRNA against CD47 (as indicated) or with scrambled control siRNA, for 48 hours. Thereafter 250,000 cells from each group were allowed to migrate through a 3 mg/ml collagen I gel and across an 8.0 µm micropore membrane for 18 hours. Cell migration was examined by staining with crystal violet blue and measuring the absorbance at 590 nm. The data are given as percent of control and represent means ± SE of five separate experiments. The statistical analyses were performed with unpaired Students t-test; *P<0.05, **P<0.01 relative to the control.

Our current finding that CD47 participates in the regulation of intestinal cell adhesion onto collagen I in addition to our previous observation that α2β1 integrin signalling leads to COX-2 expression and increased migration of intestinal epithelial cells [10], prompted us to investigate the role of CD47 in the regulation of cell migration. To do this a wound-healing assay was used to assess intestinal epithelial cell migration. This assay has the advantage of requiring neither disturbance of cell-cell adhesions nor cell detachment from the culture surface. We could show that pre-incubation with either the CD47 functional blocking antibody or the COX-2 specific inhibitor NS-398, reduced the invasive capacity of the cells by approximately 50% relative to the control, over the same time period (18 hours). Cells that were pre-incubated with a control IgG antibody or an antibody against β2 integrins (not present in these cells) were unaffected (Fig. 5A). To simulate more closely the three dimensional *in vivo* environment, cells were also allowed to migrate through a collagen I containing gel. The same pre-treatments were applied as in the wound-healing assay (Fig. 5) with similar results; the CD47 blocking antibody and NS-398 were able to significantly block cell migration, as compared to untreated cells, or cells pre-treated with the control antibodies (Fig. 5B). The siRNA against CD47 was also used to further strengthen the evidence for a role of CD47 in the regulation of migration. By knocking down CD47 using siRNA 3 and a combination of siRNAs 2 and 3 (as seen previously; Fig. 3C), we could effectively inhibit cell migration through the collagen I gel. We therefore conclude that CD47 participates in the regulation of collagen I-induced COX-2 expression and promotion of intestinal epithelial cell migration.

## Discussion

In this study we identify the pentameric transmembrane integrin associated protein, CD47, as a key regulator of collagen I-induced COX-2 expression in intestinal epithelial cells. We have previously demonstrated that the effect of collagen I on COX-2 expression in intestinal epithelial cells is mediated via collagen I engagement of cell surface α2β1 integrins [10]. Here we report several pieces of evidence for a regulatory role of CD47 in α2β1 integrin-induced COX-2 expression in intestinal epithelial cells. Firstly, we observed that CD47 co-immunoprecipitated with α2β1 integrins, a protein-protein interaction previously demonstrated in smooth muscle cells [21]. However, in addition to this previous observation in smooth muscle cells we observed that the association of CD47 with α2β1 integrins was significantly increased upon collagen I-induced engagement of α2β1 integrins on intestinal epithelial cells. Secondly, we also demonstrated the involvement of CD47 in collagen I-induced COX-2 expression by studying the effect of Gα-protein inhibition. The rational behind this approach was the well-known fact that integrins do not trigger G-protein activation, whereas CD47 has been shown to require a PTX sensitive G-protein to transduce its downstream effects [32]. In accordance with our hypothesis of a role for CD47 in collagen I-induced COX-2 expression we found a requirement for a PTX sensitive G-protein that we have identified as Gα_i3_. Indeed, this is the first time that the specific G-protein employed by CD47 has been identified. Thirdly, the role of CD47 in α2β1 integrin-induced COX-2 expression in intestinal epithelial cells was also demonstrated by three distinct approaches: use of a functional blocking anti-CD47 antibody (B6H12); a dominant negative CD47 construct and finally siRNA knock down of CD47. The above experiments convincingly demonstrate the regulatory role of CD47 in α2β1 integrin-induced COX-2 expression and provide an additional piece of important information: namely, that the association between α2β1 integrin and CD47 most certainly occurs prior to the initiation of downstream Gα_i3_-protein activation as evident from the lack of effect of PTX.

Considering our present finding that CD47 participates in the regulation of α2β1 integrin-induced COX-2 expression and previous data implicating COX-2 as a key regulator of intestinal epithelial cell migration [10], it is reasonable to suggest that CD47 participates in the regulation of intestinal epithelial cell migration [33]. Indeed, a role for CD47 in the regulation of cell motility has been documented in macrophage phagocytosis [26] and smooth muscle cells [20]. In the present study we show that an anti-CD47 blocking antibody, known to inhibit COX-2 expression, decreased migration of intestinal epithelial cells to a similar extent as the selective COX-2 inhibitor NS-398. In contrast to the previously documented role of CD47 in the regulation of phagocytosis and smooth muscle cell migration our results stress the role of COX-2 expression and activity in CD47 regulated intestinal epithelial cell migration. We observed similar results regarding the contribution of CD47 and COX-2 in intestinal cell migration in both a 2-dimensional assay on a collagen I coated surface and in a 3-dimensional assay that required migration through a collagen I containing gel. These results also led us to investigate the morphology of the migrating intestinal epithelial cells on a collagen I coated surface and how any specific morphological features may relate to CD47 and COX-2 activity.

Interestingly, we found that intestinal epithelial cells migrating on a collagen I coated surface exhibited distinct membrane blebs. Formation of non-apoptotic membrane blebs (NAMB) has been shown to be involved in an amoeboid type of neutrophil migration and migration of certain cancer cells as a means for them to invade through extracellular matrixes [34], [35]. We hypothesise that the structures we observed are indeed NAMB and that they are driving CD47 and α2β1 integrin dependent intestinal epithelial cell migration. These assumptions are supported by the findings that Rho A and F-actin were enriched in these membrane bleb structures, which is in agreement with the previous demonstration that Rho A participates in the control of NAMB-dependent cell migration [36]. In addition, cells using NAMB to migrate have been shown to have a reduced adhesive capacity for the matrix [37] and accordingly we observed an increase in intestinal epithelial cell adhesion in the presence of the anti-CD47 inhibitory antibody. The assumption that NAMB are driving CD47 and α2β1 integrin dependent intestinal epithelial cell migration is supported by the observation that CD47 and α2β1 integrins co-localized in the membrane blebs and inhibition of CD47 or COX-2 abolished not only intestinal epithelial cell migration but also the appearance of these membrane bleb structures.

The formation of NAMB in intestinal epithelial cells appears to be related to collagen I engagement of α2β1 integrins since NAMB were not present on cells adhering to and spreading on a fibronectin coated surface. The latter extracellular matrix protein is known to engage α5β1 and αvβ3 integrins, both of which have the capacity to associate with CD47 [21], [22]. Therefore we conclude that the formation of these NAMB and its migratory phenotype requires both the engagement of a particular integrin(s) and its association with CD47.

In summary, we have identified CD47 as a regulator of collagen I-induced COX-2 expression and intestinal epithelial cell migration. This represents a novel role for CD47 and reveals a potential role of this plasma membrane protein as a therapeutic target in the treatment of inflammatory bowel disease and colon cancer progression.
